# Why study the T1D remission phase in the pediatric population?^☆^

**DOI:** 10.1016/j.jped.2024.12.001

**Published:** 2024-12-20

**Authors:** Jesper Johannesen

**Affiliations:** a Copenhagen University Hospital, Herlev, Denmark; b Steno Diabetes Center Copenhagen, Copenhagen, Denmark; c University of Copenhagen, Department of Clinical Medicine, Copenhagen, Denmark

Childhood-onset type 1 diabetes (T1D) is a severe disease, not only in terms of disabling late complications leading to shortened life expectancy but also in terms of the heavy burden on the patients and their families due to an extremely demanding therapy. The etiology of T1D is, in spite of intensive biochemical, immunological, epidemiological, and clinical research during the last 100 years, still unknown. T1D seems to be a result of a complex interplay between genetic predisposition, the immune system, and environmental factors^1^^,^^2^ causing attrition and death of the insulin-producing pancreatic β cells, resulting in a life-long requirement for exogenous insulin. The progressive loss of β cells is mainly caused by autoimmune inflammation.

For decades we had treated T1D solely as an endocrine condition by various insulin substitution regimens going from pen treatment to insulin pump systems and most recently to AID systems with increasing success by obtaining glucose metabolism closer to the near-normal range.

However, maintained endogenous insulin production (measured by serum C-peptide) seems most important and adds to optimal blood glucose regulation and reduces the risk of late diabetes complications and premature death.^3^ Recently, this effect was confirmed in a large representative cohort suggesting that even minimal residual C-peptide secretion could have major clinical benefit in T1D.^4^ These observations have over the last decade led to the acceptance, that preserving beta-cell function by beta-cell protective mechanisms or immune modulating strategies will have a place to ensure better long-term outcomes and exploit that T1D is both an autoimmune and endocrine condition.

Hence, the exploration of the natural history of the T1D remission phase has drawn increasing attention over the last few years. Full remission is defined as no exogenous insulin administration and normal glucose metabolism is rarely seen and almost never in the pediatric population, whereas various definitions of partial remission have been proposed. As described in the paper of Ramos et al.^5^ in the current issue of JPED these definitions are all strongly associated, as they all include HbA1c and TDD insulin requirements in various combinations. Studies from various centers across the world are important to enlighten various factors that locally may influence the remission phase, as an in-depth understanding hereof serves as the basis for personalized putative intervention strategies.

Furthermore, it has been increasingly clear that T1D is a much more heterogenic condition than initially anticipated which also is reflected within the remission phase. A recent study from INNODIA demonstrated fasting C-peptide increased with age and over time C-peptide remained lower in younger age although a decline in C-peptide was demonstrated in all age groups.^6^ Lower baseline fasting C-peptide, BMI SD score, and presence of diabetic ketoacidosis at diagnosis were associated with lower stimulated C-peptide over time.^6^ Insulin sensitivity during the remission phase also seems to vary between individuals and influence the metabolic outcome, however, more studies are needed.^7^

The first proof of concept studies indicating that immunotherapy could be a way of preserving β-cell function came from the use of cyclosporine in new-onset T1D, which was first tested in the 1980s and successfully prolonged the remission phase.^8^ However, due to severe side effects, mainly nephrotoxicity, the use of cyclosporine was ceased. Later on, anti-lymphocyte globulin and small molecules (cyclosporine, azathioprine, and glucocorticoids) were commonly used in a regimen as a means of nonspecific immunosuppression for β-cell preservation in individuals with T1D or in islet transplantation.^9^ While glucocorticoids are widely used as an immunosuppressive steroid to treat autoimmunity^10^ it is increasingly clear that glucocorticoids adversely stimulate gluconeogenesis in the liver and antagonize the insulin-mediated uptake of glucose.^11^

Today most immunotherapies in T1D are based on the known pathogenetic mechanisms underlying the development of the disease. These targeted therapies can broadly be divided into non-antigen or antigen-specific intervention strategies, the former includes T-cell and B-cell as well as anti-cytokine targeting modalities.^12^

Recently, strategies focusing upon beta-cell rescue by anti-viral treatment^13^ and beta-cell protection by verapamil^14^ have demonstrated higher stimulated-peptide levels compared to placebo 12 months post-diagnosis. However, the current status of various intervention therapies shortly after the clinical onset of T1D demonstrates at best a temporary effect and the long-term outcome is still unsatisfactory. This may be related to various factors, such as the design and timing of the intervention, the target of modulation, and whom to target. Most of the studies today have focused on individuals with newly onset T1D, testing a single drug selected based on a pathogenetic model of the development of T1D in a predefined time span with endogenous secreted C-peptide as the primary endpoint. Increasing data are emerging so that this could turn out to be a too simplistic approach. As demonstrated, accumulating evidence demonstrate that T1D is much more heterogeneous than previously assumed which should be reflected in future preventive strategies of T1D. Further, as not all participants in the preventative T1D trials have benefitted from the tested intervention, new strategies to identify responders vs. non-responders are urgently needed and hence, development of better biomarkers is warranted.^15^ Also, further characterization of immune phenotypes seems of importance in relation to outcome.^16^

## Conflicts of interest

The authors declare no conflicts of interest.

## References

[1] Paul D.S., Teschendorff A.E., Dang M.A., Lowe R., Hawa M.I., Ecker S. (2016). Increased DNA methylation variability in type 1 diabetes across three immune effector cell types. Nat Commun.

[2] Rewers M., Ludvigsson J. (2016). Environmental risk factors for type 1 diabetes. Lancet.

[3] Nathan D.M., Genuth S., Lachin J., Cleary P., Crofford O., Diabetes Control and Complications Trial Research Group (1993). The effect of intensive treatment of diabetes on the development and progression of long-term complications in insulin-dependent diabetes mellitus. N Engl J Med.

[4] Jeyam A., Colhoun H., McGurnaghan S., Blackbourn L., McDonald T.J., Palmer C.N. (2021). Clinical Impact of Residual C-Peptide Secretion in Type 1 Diabetes on Glycemia and Microvascular Complications. Diabetes Care.

[5] Ramos M.E.N., Leão I.S., Vezzani J.R.D., Campos L.N.R., Luescher J.L., Berardo R.S. (2025). An analysis of the remission phase in type 1 diabetes within a multiethnic Brazilian sample. J Pediatr (Rio J).

[6] Marcovecchio M.L., Hendriks A.E., Delfin C., Battelino T., Danne T., Evans M.L. (2024). The INNODIA Type 1 Diabetes Natural History Study: a European cohort of newly diagnosed children, adolescents and adults. Diabetologia.

[7] Mørk F.C., Madsen J.O., Jensen A.K., Hall G.V., Pilgaard K.A., Pociot F. (2022). Differences in insulin sensitivity in the partial remission phase of childhood type 1 diabetes; a longitudinal cohort study. Diabet Med.

[8] Stiller C.R., Dupre J., Gent M., Heinrichs D., Jenner M.R., Keown P.A. (1987). Effects of cyclosporine in recent-onset juvenile type 1 diabetes: impact of age and duration of disease. J Pediatr.

[9] Shapiro A.M., Lakey J.R., Ryan E.A., Korbutt G.S., Toth E., Warnock G.L. (2000). Islet transplantation in seven patients with type 1 diabetes mellitus using a glucocorticoid-free immunosuppressive regimen. N Engl J Med.

[10] Kuo T., McQueen A., Chen T.C., Wang J.C. (2015). Regulation of Glucose Homeostasis by Glucocorticoids. Adv Exp Med Biol.

[11] Coutinho A.E., Chapman K.E. (2011). The anti-inflammatory and immunosuppressive effects of glucocorticoids, recent developments and mechanistic insights. Mol Cell Endocrinol.

[12] Johannesen J., Pociot F., Holt RI, Flyvbjerg A (2022). Textbook of Diabetes.

[13] Krogvold L., Mynarek I.M., Ponzi E., Mørk F.B., Hessel T.W., Roald T. (2023). Pleconaril and ribavirin in new-onset type 1 diabetes: a phase 2 randomized trial. Nat Med.

[14] Forlenza G.P., McVean J., Beck R.W., Bauza C., Bailey R., Buckingham B. (2023). Effect of verapamil on pancreatic beta cell function in newly diagnosed pediatric type 1 diabetes: a randomized clinical trial. JAMA.

[15] Wherrett D.K., Chiang J.L., Delamater A.M., DiMeglio L.A., Gitelman S.E., Gottlieb P.A. (2015). Defining pathways for development of disease-modifying therapies in children with type 1 diabetes: a consensus report. Diabetes Care.

[16] Dufort M.J., Greenbaum C.J., Speake C., Linsley P.S. (2019). Cell type-specific immune phenotypes predict loss of insulin secretion in new-onset type 1 diabetes. JCI Insight.

